# Prevalence and risk factors associated with HIV and syphilis co-infection in the African Cohort Study: a cross-sectional study

**DOI:** 10.1186/s12879-021-06668-6

**Published:** 2021-10-30

**Authors:** Laura Gilbert, Nicole Dear, Allahna Esber, Michael Iroezindu, Emmanuel Bahemana, Hannah Kibuuka, John Owuoth, Jonah Maswai, Trevor A. Crowell, Christina S. Polyak, Julie A. Ake, the AFRICOS Study Group, Danielle Bartolanzo, Alexus Reynolds, Katherine Song, Mark Milazzo, Leilani Francisco, Shauna Mankiewicz, Steven Schech, Alexandra Golway, Badryah Omar, Tsedal Mebrahtu, Elizabeth Lee, Kimberly Bohince, Ajay Parikh, Jaclyn Hern, Emma Duff, Kara Lombardi, Michelle Imbach, Leigh Anne Eller, Hannah Kibuuka, Michael Semwogerere, Prossy Naluyima, Godfrey Zziwa, Allan Tindikahwa, Hilda Mutebe, Cate Kafeero, Enos Baghendaghe, William Lwebuge, Freddie Ssentogo, Hellen Birungi, Josephine Tegamanyi, Paul Wangiri, Christine Nabanoba, Phiona Namulondo, Richard Tumusiime, Ezra Musingye, Christina Nanteza, Joseph Wandege, Michael Waiswa, Evelyn Najjuma, Olive Maggaga, Isaac Kato Kenoly, Barbara Mukanza, Jonah Maswai, Rither Langat, Aaron Ngeno, Lucy Korir, Raphael Langat, Francis Opiyo, Alex Kasembeli, Christopher Ochieng, Japhet Towett, Jane Kimetto, Brighton Omondi, Mary Leelgo, Michael Obonyo, Linner Rotich, Enock Tonui, Ella Chelangat, Joan Kapkiai, Salome Wangare, Zeddy Bett Kesi, Janet Ngeno, Edwin Langat, Kennedy Labosso, Joshua Rotich, Leonard Cheruiyot, Enock Changwony, Mike Bii, Ezekiel Chumba, Susan Ontango, Danson Gitonga, Samuel Kiprotich, Bornes Ngtech, Grace Engoke, Irene Metet, Alice Airo, Ignatius Kiptoo, John Owuoth, Valentine Sing’oei, Winne Rehema, Solomon Otieno, Celine Ogari, Elkanah Modi, Oscar Adimo, Charles Okwaro, Christine Lando, Margaret Onyango, Iddah Aoko, Kennedy Obambo, Joseph Meyo, George Suja, Michael Iroezindu, Yakubu Adamu, Nnamdi Azuakola, Mfreke Asuquo, Abdulwasiu Bolaji Tiamiyu, Afoke Kokogho, Samirah Sani Mohammed, Ifeanyi Okoye, Sunday Odeyemi, Aminu Suleiman, Lawrence Umejo, Onome Enas, Miriam Mbachu, Ijeoma Chigbu-Ukaegbu, Wilson Adai, Felicia Anayochukwu Odo, Rabi Abdu, Rosemary Akiga, Helen Nwandu, CHisara Okolo, Ndubuisis Okeke, Zahra Parker, Asogwa Ugochukwu Linus, Concilia Amaka Agbaim, Tunde Adegbite, Nkenchiere Harrison, Adewale Adelakun, Ekeocha Chioma, Victoria Idi, Rachel Eluwa, Jumoke Nwalozie, Igiri Faith, Blessing Okanigbuan, Achugwo Emmanuel, Nkiru Nnadi, Ndubuisi Rosemary, Uzoegwu Amaka Natalie, Obende Theresa Owanza, Falaju Idowu Francis, Jacintal Elemere, Obilor Ifeoma Lauretta, Edward Akinwale, Inalegwu Ochai, Lucas Maganga, Emmanuel Bahemana, Samoel Khamadi, John Njegite, Connie Lueer, Abisai Kisinda, Jaquiline Mwamwaja, Faraja Mbwayu, Gloria David, Mtasi Mwaipopo, Reginald Gervas, Doroth Mkondoo, Nancy Somi, Paschal Kiliba, Gwamaka Mwaisanga, Johnisius Msigwa, Hawa Mfumbulwa, Peter Edwin, Willyhelmina Olomi

**Affiliations:** 1 U.S. Military HIV Research Program, Walter Reed Army Institute of Research, Silver Spring, MD USA; 2 Henry M. Jackson Foundation for the Advancement of Military Medicine Inc., Bethesda, MD USA; 3 HJF Medical Research International, Abuja, Nigeria; 4 HJF Medical Research International, Mbeya, Tanzania; 5 Makerere University-Walter Reed Project, Kampala, Uganda; 6 U.S. Army Medical Research Directorate – Africa, Kisumu, Kenya; 7 HJF Medical Research International, Kisumu, Kenya; 8 HJF Medical Research International, Kericho, Kenya

**Keywords:** Human immunodeficiency virus (HIV), Syphilis, Africa

## Abstract

**Background:**

Each year, 5.6 million new syphilis cases are diagnosed globally. Guidelines for people living with HIV (PLWH) in low-income countries (LIC) recommend STI testing for symptomatic persons and those newly diagnosed with HIV; routine STI testing is less clear. Here we provide updated syphilis prevalence and identify co-infection risk factors in PLWH in the African Cohort Study (AFRICOS) to understand these rates as they relate to syndromic treatment.

**Methods:**

AFRICOS is a study enrolling PLWH and HIV-uninfected individuals in four African countries. Participant study enrollment information was used to determine syphilis prevalence and co-infection risk factors. Inclusion criteria consisted of adults 18 years or older receiving care at a participating clinic as a long-term resident who consented to data and specimen collection. Exclusion criteria consisted of pregnancy and/or imprisonment. Screen-positive syphilis was defined as a reactive rapid plasma regain (RPR) upon study enrollment whereas confirmed syphilis included a reactive RPR followed by reactive treponemal test. Multivariate analyses was performed to determine HIV and syphilis co-infection risk factors.

**Results:**

Between 2013 and March 1, 2020, 2939 PLWH enrolled and 2818 were included for analysis. Screen-positive and confirmed syphilis prevalence were 5.3% (151/2818) and 3.1% (87/2818), respectively. When the analysis was restricted to PLWH with an RPR titer of greater than, or equal to, 1:8, 11/87 (12.6%) participants were included. No PLWH and confirmed syphilis had documented genital ulcers. In the multivariate model, participants with confirmed syphilis co-infection were more likely to have none or some primary education [aOR 3.29 (1.60, 6.74)] and consume alcohol [aOR 1.87 (1.16, 3.03)] compared to those without syphilis. Antiretroviral therapy (ART) with suppressed viral load (VL) was protective in the unadjusted model but not adjusted multivariate model.

**Conclusions:**

Our findings show that syphilis rates in sub-Saharan Africa remain elevated where diagnosis remains challenging, and that both lower education level and alcohol consumption are significantly associated with HIV/syphilis co-infection in AFRICOS. Based on our analysis, current STI guidelines targeting testing for African individuals with either new HIV diagnosis or syndromic symptoms may be inadequate, highlighting the need for increased testing and treatment strategies in resource-limited settings.

## Background

Every year, 5.6 million new syphilis cases are diagnosed in men and women ages 15–49 years old globally [1, 2]. Comprehensive studies on syphilis prevalence in low-income countries (LIC) are mostly extrapolated from antenatal care clinics where prevalence is estimated at 3.5–4.6% in Africa (excluding South Africa) [2, 3]. In PLWH, a population where co-infections with sexually transmitted infections (STIs) frequently coexist due to their shared mode of sexual transmission, few studies have researched syphilis prevalence in LIC through examination of serologic confirmatory testing, largely due to diagnostic challenges. Capturing an accurate assessment of syphilis prevalence in PLWH is of particular public health concern because it not only increases the likelihood of HIV transmission, but also alters the natural history of HIV, where PLWH experience a temporary reduction in CD4 counts and elevation in HIV viral load (VL) in early, untreated syphilis infections [4]. Besides the epidemiological aspect, the co-existence of these two infectious diseases is also of clinical and diagnostic interest: PLWH more commonly demonstrate atypical clinical features of syphilis and serological interpretation of syphilis testing may not correlate with clinical presentation in this population [5].

Current World Health Organization (WHO) guidelines for PLWH living in resource-limited settings (published in 2008) recommend STI testing only for symptomatic individuals as well as those with a new HIV diagnosis, possibly leading to a large proportion of undiagnosed STIs and continued community spread [6, 7]. For treatment of syphilis, the WHO recommends intramuscular benzathine penicillin (one dose in early syphilis and once weekly for three consecutive weeks in late or unknown syphilis) [8]. Routine STI testing is less clear and thus, updated data on HIV and syphilis co-infection prevalence and risk factors is urgently needed in order to not only understand these rates as they are related to using syndromic treatment but also to guide newer recommendations for PLWH, especially if Africa is to attain the goal of 90% reduction of syphilis by 2030 as outlined by the WHO [2]. Here we provide epidemiologic information and describe significant risk factors for PLWH and serologically confirmed syphilis co-infection in the African Cohort Study (AFRICOS).

## Methods

### Description of AFRICOS Cohort

AFRICOS is an ongoing longitudinal study enrolling PLWH and HIV-uninfected individuals at 12 large public and private health facilities supported through the US Military HIV Research Program (MHRP) by the President's Emergency Plan for AIDS Relief (PEPFAR) in Kenya, Tanzania, Uganda, and Nigeria. Its objective is to assess the impact of clinical practices, biological factors, and socio-behavioral issues on HIV infection and disease progression in African settings with aims of informing practice and policy [9]. Enrollment began in January 2013. Individuals were eligible if they were 18 years or older, receiving care at an enrolling PEPFAR clinic, intended to be a long-term resident of the area, willing to provide demographic information and be contacted by the study staff, and consented to data and specimen collection. Exclusion criteria consisted of any significant condition that in the opinion of the study investigator might interfere with the conduct of the study, pregnancy, or if the subject was a prisoner [9]. All participants provided written informed consent. The study was approved by institutional review boards of the Walter Reed Army Institute of Research, Makerere University School of Public Health, Kenya Medical Research Institute, Tanzania National Institute of Medical Research, and the Nigerian Ministry of Defense [9].

Demographic, laboratory (including HIV-specific and syphilis testing) as well as clinical information (including physical exam) is captured upon AFRICOS enrollment and at each bi-annual visit for every AFRICOS participant. For this cross-sectional study, participant information gathered at time of AFRICOS enrollment was used to determine syphilis screen-positive and confirmed syphilis prevalence, as well as risk factors for HIV and syphilis co-infection. Because untreated syphilis is associated with long-term neurologic sequelae, cognitive impairment is also assessed in AFRICOS participants. Measurement of cognitive impairment upon AFRICOS enrollment was with the International HIV Dementia Scale (IHDS), which includes evaluation of memory recall, motor speed and psychomotor speed [10].

### Study enrollment questionnaire

Risk factors for HIV and syphilis co-infection were ascertained through examination of study participants’ responses to a comprehensive behavioral questionnaire upon AFRICOS enrollment. The questionnaire included information regarding demographics (including education history), sexual history and behaviors, social history and behaviors (including substance abuse), general health and medical conditions, as well as cognition. Education was categorically defined by participants selecting their highest level of education completed. Alcohol consumption was dichotomously defined as either “Yes” or “No” in response to the question: “Do you consume alcohol?”.

### Syphilis diagnosis

All countries utilize the rapid plasma reagin (RPR) as the initial (non-treponemal) screening test followed by a confirmatory (treponemal) test, where most countries use the *Treponema pallidum* antibody (TPA) as the treponemal test. Screen-positive syphilis was defined as having only a reactive RPR upon study enrollment, where any RPR titer that was greater than, or equal to, 1:2 was included in the analysis. Serologically-confirmed syphilis was defined as having a reactive RPR as well as a reactive treponemal test upon study enrollment. AFRICOS participants with a diagnosis of serologically-confirmed syphilis and a reactive RPR titer equal to, or greater than, 1:8 were further analyzed given their higher likelihood of having an active syphilis infection. All countries in this study utilized the traditional syphilis diagnostic algorithm (no countries utilized the reverse syphilis algorithm). Syphilis diagnostic testing was performed in an AFRICOS-associated laboratory and no point of care (POC) syphilis testing was included in this analysis.

### Statistical design

Bivariate analyses using χ2 tests for categorical variables were conducted to compare cases of confirmed syphilis by demographic, clinical, and socio-behavioral factors. All the independent variables investigated in our analyses had less than 6% missing data. Logistic regression models were used to estimate unadjusted and adjusted odds ratios and 95% confidence intervals (95% CIs) for associations between various factors and confirmed syphilis. Factors with a p-value < 0.05 from the bivariate analysis were included in the multivariate model along with additional factors identified a priori as being relevant to syphilis. Analyses were performed in SAS version 9.3 (SAS Institute, Cary, North Carolina) and Stata version 15.0 (StataCorp, College Station, Texas) software.

## Results

### Characteristics of all AFRICOS participants

Between January 21, 2013 and March 1, 2020, 2939 PLWH were enrolled into AFRICOS and 2818 had complete covariate and outcome data and were included in this analysis. A total of 1649/2818 (58.5%) of participants were female and median age was 38.3 (Interquartile range, IQR: 31, 46) years old. The highest enrollment site was South Rift Valley, Kenya (36% of participants) and most frequent education category was primary or some secondary (39% of participants). A total of 1986/2818 (70%) participants were taking antiretroviral therapy (ART) upon study enrollment where the median time since HIV diagnosis was 2.5 (IQR: 0.2–6.3) years and the median time on ART was 3.0 (IQR: 0.7–5.9) years. Median CD4 count for all PLWH enrolled was 390 (IQR: 238, 573) cells/mm^3^ and 1361/2818 (48.3%) had a HIV viral load less than 50 copies/mL while taking ART (Table 1).

**Table 1:** Baseline characteristics of PLWH and syphilis co-infection

	All (n = 2818)	No syphilis (n = 2731)	Syphilis (n = 87)	P value
Gender				0.19
Male	1169 (41.5%)	1127 (41.3%)	42 (48.3%)	
Female	1649 (58.5%)	1604 (58.7%)	45 (51.7%)	
Study site				< 0.001
Kayunga, Uganda	515 (18.3%)	496 (18.2%)	19 (21.8%)	
South Rift Valley, Kenya	1007 (35.7%)	977 (35.8%)	30 (34.5%)	
Kisumu West, Kenya	499 (17.7%)	499 (18.3%)	0	
Mbeya, Tanzania	495 (17.6%)	457 (16.7%)	38 (43.7%)	
Abuja & Lagos Nigeria	302 (10.7%)	302 (11.1%)	0	
Age at visit				0.13
18–29	506 (18.0%)	496 (18.2%)	10 (11.5%)	
30–39	982 (34.8%)	947 (34.7%)	35 (40.2%)	
40–49	819 (29.1%)	788 (28.9%)	31 (35.6%)	
50+	511 (18.1%)	500 (18.3%)	11 (12.6%)	
Age at visit^a^	38.3 (31.2, 46.0)	38.3 (31.2, 46.0)	38.4 (31.7, 45)	0.92
CD4 count (cells/uL)^a^	390 (238, 573)	393 (239, 575)	323 (182, 456)	0.006
CD4 count (cells/uL)				0.037
Less than 200	549 (19.5%)	526 (19.3%)	23 (26.4%)	
200–499	1313 (46.6%)	1268 (46.4%)	45 (51.7%)	
Greater than/equal to 500	956 (33.9%)	937 (34.3%)	19 (21.8%)	
Viral load (copies/mL)				
Less than 50	1361 (48.3%)	1327 (48.6%)	34 (39.1%)	< 0.001
Greater than 50	588 (20.9%)	574 (21%)	14 (16.1%)	
Not on ART	869 (30.8%)	830 (30.4%)	39 (44.8%)	
Education category				0.002
None/some primary	947 (33.6%)	908 (33.2%)	39 (44.8%)	
Primary/some secondary	1099 (39.0%)	1061 (38.9%)	38 (43.7%)	
Secondary and above	772 (27.4%)	762 (27.9%)	10 (11.5%)	
Cognitive impairment^b^				0.66
No	2658 (94.3%)	2575 (94.3%)	83 (95.4%)	
Yes	160 (5.7%)	156 (5.7%)	4 (4.6%)	
Consume alcohol				0.002
No	2277 (80.8%)	2218 (81.2%)	59 (67.8%)	
Yes	541 (19.2%)	513 (18.8%)	28 (32.2%)	
Support group				0.054
No	2467 (87.5%)	2385 (87.3%)	82 (94.3%)	
Yes	351 (12.5%)	346 (12.7%)	5 (5.7%)	
History of circumcision				0.051
Not circumcised	396 (14.1%)	376 (13.8%)	20 (23.0%)	
Circumcised	773 (27.4%)	751 (27.5%)	22 (25.3%)	

^a^Median, IQR; ^b^International HIV Dementia Scale (IHDS)

### Prevalence of screen-positive and serologically confirmed syphilis

Prevalence of screen-positive and confirmed syphilis upon AFRICOS enrollment in PLWH was 5.3% (151/2818) and 3.1% (872818), respectively. None of the PLWH and confirmed syphilis co-infection had documented genital ulcers on enrollment exam (prevalence of 1.5% among PLWH overall). When the analysis was restricted to PLWH who had an RPR titer of greater than, or equal to, 1:8, 11/87 (12.6%) participants were included. Further comparison of PLWH with confirmed syphilis among those with high and low titers was not able to be performed due to the small sample size resulting in insufficient power for analysis.

### Characteristics of PLWH and serologically confirmed syphilis

Bivariate analysis for risk factors associated with HIV and serologically confirmed syphilis co-infection was performed on enrollment data for all PLWH (Table 1). Among PLWH with serologically confirmed syphilis (n = 87), a total of 45/87 (51.7%) were female, median age was 38 (32, 45) years and 56.2% were married. Additionally, PLWH and confirmed syphilis had lower median CD4 counts (p-value < 0.01) and fewer were virally suppressed compared to those without syphilis (39.1% vs 48.6%, respectively) (p-value < 0.001). They also had a shorter duration of both HIV diagnosis and ART administration.

### Multivariate analysis

Multivariate analysis was performed to assess risk factors for PLWH and syphilis co-infection and are displayed in Table 2. In the multivariate model, statistically significant risk factors that were associated with an increased risk for HIV and syphilis co-infection included education level and alcohol consumption. Having no/some primary education (compared to PLWH and history of secondary education and above) increased the risk for HIV and syphilis co-infection [OR: 3.29; 95% CI (1.60, 6.74)]. Alcohol consumption also increased the risk for HIV and syphilis co-infection [OR: 1.87; 95% CI (1.16, 3.03)]. Analysis demonstrated that ART administration with VL less than 200 copies/mL was considered protective in the unadjusted model but did not reach significance in the adjusted model.

**Table 2:** Factors associated with syphilis co-infection among PLWH

	Unadjusted odds ratio (95% CI)	Adjusted odds ratio (95% CI)
Gender		
Female	0.75 (0.49, 1.15)	
Age (Years)		
18–29	Reference	Reference
30–39	1.83 (0.90, 3.73)	1.74 (0.85, 3.57)
40–49	1.95 (0.95, 4.02)	2.06 (0.98, 4.33)
50+	1.09 (0.46, 2.59)	1.19 (0.49, 2.93)
Education level		
None/some primary	3.27 (1.62, 6.60)	3.29 (1.60, 6.74)
Primary/some secondary	2.73 (1.35, 5.51)	2.63 (1.29, 5.37)
Secondary and above	Reference	Reference
Cognitive impairment^a^		
Yes	0.80 (0.29, 2.20)	0.70 (0.25, 1.96)
Alcohol consumption		
Yes	2.05 (1.30, 3.25)	1.87 (1.16, 3.03)
Support group involvement		
No	2.38 (0.96, 5.91)	1.89 (0.75, 4.79)
History of circumcision		
Not circumcised	1.90 (0.11, 3.25)	1.44 (0.82, 2.51)
Circumcised	Reference	Reference
Viral suppression		
On ART, VL < 200 copies/mL	0.50 (0.32, 0.80)	0.65 (0.39, 1.08)
On ART, VL > 200 copies/mL	0.66 (0.34, 1.24)	0.73 (0.38, 1.40)
Not on ART	Reference	Reference
CD4 count^b^		
Less than 200	0.81 (0.49, 1.36)	0.89 (0.52, 2.51)
200–499	0.46 (0.25, 0.86)	0.53 (0.28, 1.02)
Greater than/equal to 500	Reference	Reference

^a^International HIV Dementia Scale (IHDS); ^b^cells/uL

## Discussion

In this well-characterized cohort of PLWH in Africa, prevalence of screen-positive and confirmed syphilis co-infection was 5.3% and 3.1%, respectively, demonstrating that syphilis rates remain elevated in the African context. Our results also demonstrate the challenges in the diagnosis of syphilis infection—and suggest a role for serologic confirmatory testing, as almost half of PLWH initially diagnosed with syphilis would have received unnecessary treatment based on results of the initial (non-treponemal) screening syphilis test alone. There are many reasons for a false-positive non-treponemal test, to include a diagnosis of HIV, and these findings highlight the need for increased testing capability in resource-limited settings.

The cohort’s prevalence rates are slightly higher than prior African studies examining serologically confirmed syphilis: Djomand et al. reported an HIV and serologically confirmed syphilis co-infection rate of 0.6% in females and 2.4% in 559 newly diagnosed individuals with HIV entering care in their cross-sectional study Namibia [11]. In this study, the median age was 30 years (compared to 38 years in our study participants) and none were taking ART. The higher syphilis prevalence in our study may be associated with increased years of sexual activity and therefore longer duration of potential syphilis exposure in our cohort population. In a separate study, Singa et al. reported an overall rate of 0.5% in 1661 PLWH in Kenya [12]. This lower rate may be impacted by limited sexual risk behavior: most participants were married or cohabitating, and they reported high levels of condom use.

Notably, while prevalence of confirmed syphilis was 3.1% among PLWH, none of these participants had documented genital ulcers on exam. Only 12.6% (11/87) of the PLWH with syphilis co-infection in our study had an RPR titer of greater than, or equal to, 1:8 (which represents a higher likelihood of active syphilis infection). There are different possible reasons for this finding: it could be that many of the participants in our study had either been treated for a prior syphilis infection (and subsequently resulted in being serofast) or perhaps they were recently infected, resulting in their lower CD4 counts and higher VL. Interestingly, prior studies have shown that in PLWH, syphilis serological tests may not correlate with the clinical presentation, further confounding the correct diagnosis. Altered serological tests are also possible among PLWH co-infected with syphilis: post-treatment serological values may be higher than expected in serofast individuals or fluctuate; false-negative and delayed sero-reactive tests have been also described [5]. In the context of WHO guidelines for PLWH that recommend STI testing based on symptoms, these findings add to the currently limited data available on the prevalence of asymptomatic STIs among PLWH in Africa and further highlight the need for improved syphilis diagnostic testing and treatment algorithms in LIC, especially among PLWH [7]. These findings also reinforce the importance of initiating therapy with penicillin in PLWH when syphilis is diagnosed, even if not presenting with dermatological or systemic manifestations.

In the multivariate model, statistically significant risk factors for HIV and syphilis co-infection included no/some primary education (compared to secondary education and above) and alcohol consumption. Lower educational status as a risk factor for HIV and confirmed syphilis is consistent with prior studies where Mutagoma et al. also found five times higher rates of no/primary education in PLWH and confirmed syphilis compared to those without syphilis, demonstrating the broad public health consequences of education [13]. Additionally, ART administration with HIV-1 viral suppression was found to be protective in the unadjusted model (but not adjusted model) and these results may suggest that similar factors contribute to syphilis acquisition and poor HIV virologic outcomes.

This study has several strengths to include that it evaluates a large, well-characterized cohort with a robust population of PLWH across four African countries who sought care in a variety of public and private facilities. It leverages serologic syphilis clinical data, including screening and confirmatory testing, which is uncommonly recorded in resource-limited settings. This study also identifies several important risk factors for PLWH and serologically-confirmed syphilis co-infection in non-pregnant individuals through multivariate analysis using demographic, clinical and socio-behavioral characteristics.

There are some limitations to this study. Our findings may not be generalized to individuals in other African regions. There is also limited AFRICOS enrollment of individuals who engage in high-risk sexual activities, such as commercial sex workers and men who have sex with men for whom there is both a higher prevalence of HIV and STI co-infection as well as a distinct characterization of socio-behavioral risk factors. In this analysis, sexual behavioral risk factors (to include accessibility to free condoms, number of lifetime sexual/casual partners, sex in exchange for gifts, money, shelter, food, drugs, and favors) were examined in an earlier bivariate model but not found to be significant and thus not included in later analytic models. Given the high proportion of serofast cases, associated risk factors may have been remote to study participation, impacting our analysis’ sensitivity to detect behavior impacts on syphilis acquisition. Specifically regarding the analysis of serofast cases, our study also did not examine the correlation between AFRICOS participants’ treatment history and their serological syphilis tests, limiting the ability to fully interpret these findings. Finally, our analysis did not analyze HIV and syphilis co-infection rates over time and additional studies should examine this association longitudinally so as to better guide recommendations on comprehensive care for PLWH in Africa, especially in resource-limited settings.

## Conclusions

Our findings show that syphilis rates in sub-Saharan Africa remain elevated and that both lower education level and alcohol consumption are significantly associated with HIV/syphilis coinfection in AFRICOS. Our results also demonstrate the diagnostic challenges of syphilis infection—and suggest a role for serologic confirmatory testing, as almost half of PLWH initially diagnosed with syphilis would have received unnecessary treatment based on results of the initial (non-treponemal) screening syphilis test alone. Based on our analysis, current STI guidelines that target testing for individuals with either new HIV diagnosis or syndromic symptoms in Africa may be inadequate and highlight the need for increased testing and treatment strategies in resource-limited settings.

## Data Availability

The datasets used and analyzed during the current study are available from the corresponding author on reasonable request.

## Abbreviations

AFRICOS: African Cohort Study
ART: Antiretroviral therapy
HIV: Human immunodeficiency virus
HIV-1 VL: HIV-1 viral load
IHDS: International HIV Dementia Scale
IQR: Interquartile range
LIC: Low-income country
MHRP: US Military HIV Research Program
PEPFAR: President's Emergency Plan for AIDS Relief
PLWH: People living with HIV
POC: Point of care
RPR: Rapid plasma regain
STI: Sexually transmitted infection
TPA: *Treponema pallidum* Antibody
WHO: World Health Organization
